# Analysis of air pollution mortality in terms of life expectancy changes: relation between time series, intervention, and cohort studies

**DOI:** 10.1186/1476-069X-5-1

**Published:** 2006-02-01

**Authors:** Ari Rabl

**Affiliations:** 1 Ecole des Mines, 60 boul. St.Michel, F-75272 Paris, France

## Abstract

**Background:**

Information on life expectancy change is of great concern for policy makers, as evidenced by the discussions of the so-called "harvesting" issue (i.e. the question being, how large a loss each death corresponds to in the mortality results of time series studies).

**Methods:**

Whereas most epidemiological studies of air pollution mortality have been formulated in terms of mortality risk, this paper shows that a formulation in terms of life expectancy change is mathematically equivalent, but offers several advantages: it automatically takes into account the constraint that everybody dies exactly once, regardless of pollution; it provides a unified framework for time series, intervention studies and cohort studies; and in time series and intervention studies, it yields the life expectancy change directly as a time integral of the observed mortality rate.

**Results:**

Results are presented for life expectancy change in time series studies. Determination of the corresponding total number of attributable deaths (as opposed to the number of observed deaths) is shown to be problematic. The time variation of mortality after a change in exposure is shown to depend on the processes by which the body can repair air pollution damage, in particular on their time constants. Hypothetical results are presented for repair models that are plausible in view of the available intervention studies of air pollution and of smoking cessation. If these repair models can also be assumed for acute effects, the results of cohort studies are compatible with those of time series.

**Conclusion:**

The proposed life expectancy framework provides information on the life expectancy change in time series studies, and it clarifies the relation between the results of time series, intervention, and cohort studies.

## Background

There has been much debate about the significance of the mortality impacts (sometimes called "acute mortality") observed in time series (TS) studies, an issue often referred to as harvesting or mortality displacement (see, for example, the articles by Zeger et al [1] and Schwartz [2]). The key question is whether the observed deaths have been advanced by only a few days or whether the loss of life expectancy (LE) is much larger. This issue is crucial for the monetary valuation and for policy implications [3,4].

For a new perspective on this issue and on the relation between TS, intervention, and cohort studies, the present paper formulates the analysis directly in terms of LE change, after showing that such a formulation is mathematically equivalent to the conventional formulation, in terms of mortality risk. An LE formulation offers several advantages: it automatically accounts for the fact that everybody dies exactly once, regardless of pollution; it provides a unified framework for time series, intervention studies, and cohort studies; and it directly yields a quantity of interest to policy makers.

The constraint of fixed total probability of death can be appreciated by comparing an accident that instantly kills individuals in normal health (the LE loss ΔL is equal to the entire remaining LE) with a mortality risk that reduces LE by a short amount of time ΔL. The time dependence of the mortality rates is different. Whereas for an accident, the mortality rate changes only at the moment of the accident, for the risk with the short ΔL the mortality rate increases initially but then decreases (relative to a reference population without the risk) during the ensuing period ΔL, because of the individuals who would have died then, but whose deaths were advanced. The delayed decrease can be called "compensating change". Even though TS studies up until now have not taken this constraint into account, it has not affected the results. For TS, the compensating change becomes a more or less uniform background as a result of the fluctuations in concentration because there is a wide range of individual ΔL. In cohort studies, the constraint is implicit in the study design, because they observe the net effect of chronic exposure. The constraint is also crucial for understanding the LE change in TS studies (see references 5, 6, for example) and in intervention studies [7-10].

A unified framework for TS and cohort studies has also been recently proposed by Burnett et al [11], who show that both types of studies measure essentially the same risk function. However, these authors do not take into account the time variation of the risk function, due to the compensating change (i.e. that the increased mortality due to a pollution peak now implies a decreased mortality at a later time).

The present paper shows that the mortality fluctuations observed in TS studies are proportional to the instantaneous time derivative of the life expectancy. They are the result of exposures both in the recent and the distant past, but a strong correlation with the most recent exposure is observed, since the fluctuations due to past exposures tend to average out to zero. The acute LE loss, due to a pollution peak, can be calculated by integrating the mortality rate over the observation window of a TS study (typically 1 day) and results are presented for O_3 _and PM_10 _(including studies that have extended the observation window to 60 days). In intervention studies, the (approximately) constant difference between exposures before and after the intervention makes it possible to determine the LE change by integrating the change of the mortality rate over time. Cohort studies [12-14] measure a long term relative risk from which one can calculate [15-18] the ultimate LE gain that can be achieved by a permanent reduction of air pollution; it is equal to the LE change at the end of a sufficiently long intervention study.

The determination of deaths that can be attributed to air pollution is also addressed. By contrast to the acute LE loss due to a pollution peak, the corresponding total number of deaths (in the sense of all deaths that are advanced by the peak) cannot be measured by epidemiology. The number of deaths implied by TS studies has usually been calculated by multiplying mortality rate and relative risk increase, but as shown here, that yields only a lower bound. This also prevents the determination of the LE loss per air pollution death.

Unfortunately, the available data are not sufficient to determine all quantities of interest (for example, the relation between the results of TS, intervention, and cohort studies, and the contribution of acute mortality to the total LE loss from chronic exposure). Since one needs models for the processes by which the body repairs air pollution damage, the remaining sections of the paper are somewhat speculative. Whereas the phenomenon of repair is well documented by studies of smoking cessation [19-21], less is known about repair of air pollution damage [22]. In view of the available information, it is plausible to assume that the LE change due to air pollution is proportional to the time integral of past concentrations weighted by exponential decay factors. Using model parameters suggested by the data, results are plotted for the evolution of mortality after an intervention. They indicate that the change in mortality rate is largest soon after the intervention. After a time period longer than the longest time constant of the repair processes, the mortality rate returns to a level close to the one before the intervention (even though the LE gain is permanent), a consequence of the fact that everybody will die sooner or later.

With these models, the results of TS, intervention, and cohort studies are remarkably compatible with each other. The contribution of acute mortality to the total LE loss of chronic exposure is equal to the relative risk increase times the time constant of the repair processes that are significant immediately after a pollution peak.

## Methods

### A qualitative model for effects of air pollution

Discussions of acute mortality impacts are often phrased in terms of a pool of individuals who are so frail that they succumb to a pollution peak. A large stationary population always contains many individuals who are so frail that an additional stress imposed by an air pollution peak can advance their death. For example, at any moment, roughly 1% of a stationary population with life expectancy 75 years are within the last nine months of their life, and thus extremely frail. Illness can cause temporary episodes of frailty.

**Figure 1:**
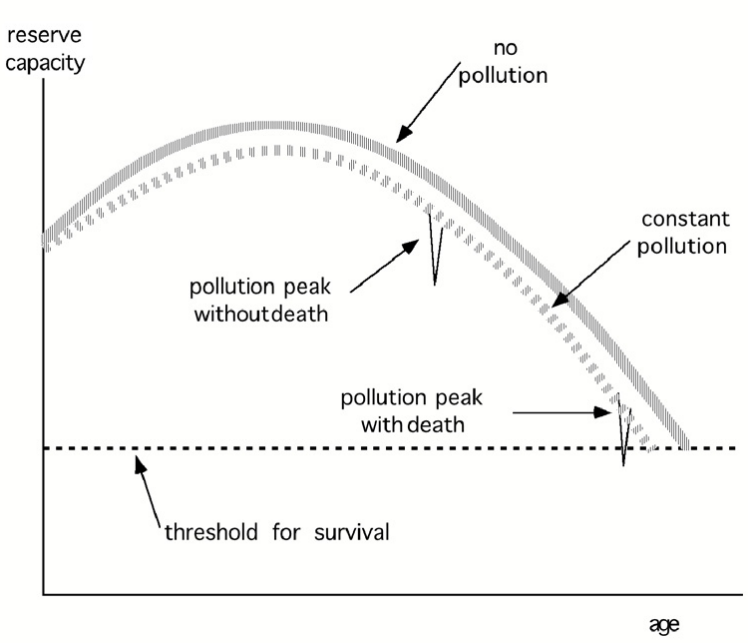
**Sketch of reserve capacity of the body. **This diagram shows, in a qualitative manner, the reserve capacity of the body as function of age and the effect of air pollution. There are natural fluctuations, for example due to illness, as suggested by the width of the lines. Air pollution lowers the curve. The effect of pollution on mortality (shown exaggerated for the sake of illustration) becomes observable in an epidemiological study only to the extent that there are individuals whose reserve capacity is so low that the extra stress pushes them below the threshold for survival.

But the fact that pollution-related deaths occur only in the frail pool does not mean that pollution has no effect on the rest of the population. Rather it contributes to reducing the reserve capacity of the body, as illustrated very schematically in Fig. 1, without trying to give a precise definition of reserve capacity other than saying that it is inversely related to frailty (Fig. 1 is inspired by a graph in chapter 4 of NRC [23]). A young, healthy body has enough reserve capacity not to feel the relatively slight reduction due to acute or chronic pollution exposure. But the old or sick may be pushed below the threshold where death occurs. As individuals age, they inevitably move into the pool of the frail. By diminishing the reserve capacity, pollution advances the passage into the frail pool and shortens life expectancy. On average, the inflow to the frail pool equals the outflow. Time series studies measure the effect of pollution fluctuations on the outflow from this pool, as reflected in the number of deaths per day.

### Change of mortality after change of exposure

**Figure 2:**
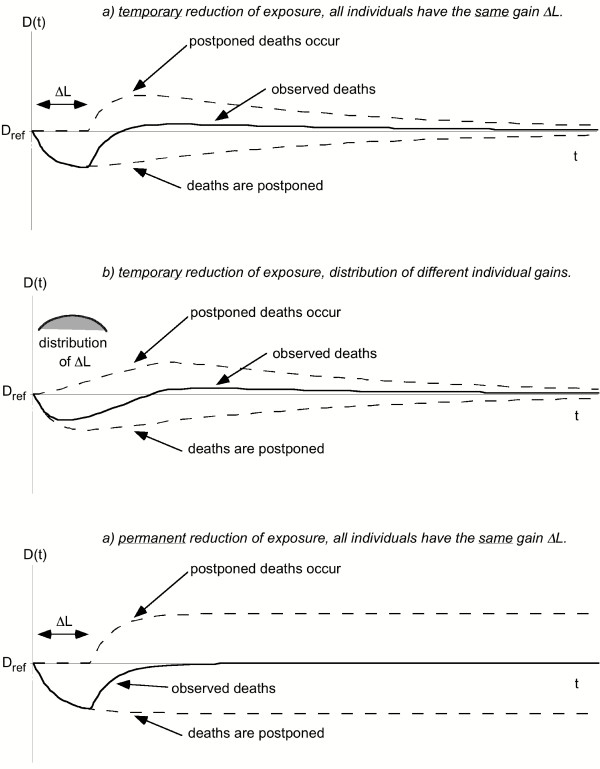
**Qualitative picture of rate of deaths after an exposure reduction. **The number of deaths per day, D, is shown versus time after the reduction. Thick solid line = observable deaths, thin dashed line = deaths shifted by pollution. a) temporary reduction of exposure, all individuals have the same gain ΔL. b) temporary reduction of exposure, distribution of different individual gains. c) permanent reduction of exposure, all individuals have the same gain ΔL.

Fig. 2 shows a qualitative picture of the effect of a reduction of exposure on D, the number of deaths per day in a stationary population. In these graphs, the lower dashed line represents the individuals whose deaths are postponed and the upper dashed line the postponed deaths when they do occur. The dashed lines are not observable. Only the net effect can be observed, shown by the heavy solid line, which is the sum of these dashed lines. The exposure reduction is temporary in parts a) and b), and permanent in c). In part a), all individuals enjoy the same LE gain ΔL, so the upper dashed line is the mirror image of the lower one, but shifted by ΔL. In part b), there is a distribution of different LE gains; it broadens the upper dashed curve. After the initial drop, the solid line moves above D_ref_; that is a manifestation of the compensating change mentioned in the introduction. The onset of the compensating change gives a rough indication of ΔL.

Part c) shows the effect of a permanent decrease of exposure (for simplicity for the case where all individuals enjoy the same LE gain ΔL). Here the postponed deaths reach a constant asymptotic level. The observable death rate drops at first, but then increases again, gradually reaching the original level, even though the LE gain is permanent. D must eventually return to the original level, because in a stationary population the birth rate is constant and equal to D. Deaths have been postponed, but not avoided. Eventually, a new stationary state is reached, with a longer life expectancy and thus a larger population (for the same birth rate). The mortality rate is the ratio of D and population size. The latter increases permanently, whereas D returns to the original value.

### Relation between age-specific mortality and life expectancy (LE)

To obtain quantitative results, it is helpful to recall some well-known elements of survival analysis. Let μ(x) be the age-specific mortality rate, defined such that someone who has reached age x has a probability μ(x) Δx of dying between x and x + Δx (usually one chooses Δx = 1 yr). The fraction of a birth cohort of initial age x_0 _that survives to age x is called survival function S_μ_(x_0_, x). As shown in Appendix A [see Additional file 1], it is given by



and the remaining LE of a cohort of age x_0 _can be calculated as



If μ(x) is given, S_μ_(x_0_, x) and L(x_0_) are thus uniquely determined. Vice versa, the function L(x) determines μ(x), as shown in Appendix A. Because of the one-to-one relationship between μ(x) and life expectancy L(x), the mortality impact of air pollution can be analyzed in terms of a change in mortality rate or in terms of the corresponding LE change ΔL. Here, and throughout the paper, ΔL is the change per person, averaged over the population or population segment under consideration.

### Relation between LE change and mortality after intervention

Let us evaluate the change in mortality and LE as a function of time t after a permanent reduction of exposure at t = 0, the population having been stationary before the intervention. Here, we look only at the entire population; more detailed equations for the effect on a cohort of a given age are derived in Appendix B [see Additional file 2]. Consider a large stationary population of N individuals and the number of deaths per time, designated by D. It will be convenient to look at D, because its change can be related directly to the postponement of the individual deaths. The population-averaged mortality rate μ (i.e. the average of μ(x) over the age distribution) is the ratio

μ = D/N.     (3)

Let μ_ref _be the mortality before and μ(t) be the mortality after an intervention that reduces air pollution permanently by a constant amount. The relative risk is

RR(t) = μ(t)/μ_ref _    (4)

and it corresponds to an LE change

ΔLt) = L(t) - L_ref_.     (5)

D and N are functions of the time t after the intervention. D and N before the intervention, designated by D_ref _and N_ref_, are independent of time, because the population is stationary. D_ref _is equal to the birth rate. To find the evolution of D(t) and N(t) after the intervention and the relation between μ(t) and ΔL(t), let us begin by assuming a homogeneous population in the sense that all individuals experience the same LE gain ΔL(t).

**Figure 3:**
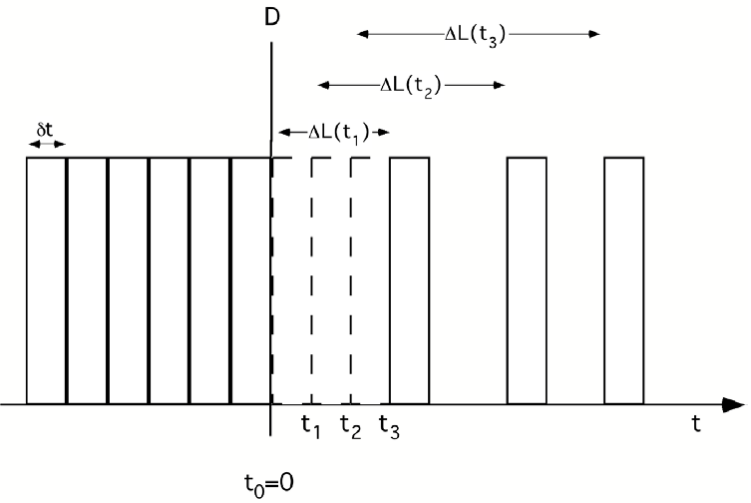
**Derivation of rate D of deaths per time after intervention. **This sketch illustrates the derivation of the evolution of the rate D of deaths per time after a step decrease of pollution. D is proportional to the density of boxes, each box representing the deaths per small time step δt. After exposure reduction the deaths are delayed and the boxes shifted to the right. The dashed boxes show from where the boxes are shifted by the intervention. ΔL(t) = LE gain at time t after intervention.

It is helpful to consider small discrete time steps δt and to plot D as a sequence of boxes of width δt, with the height representing the number of deaths during δt (see Fig. 3). Before the intervention, D = D_ref _= constant and the spacing of the boxes is uniform. After the intervention, the deaths occur later. The effect of this postponement of deaths on D can easily be understood by considering the shift of the boxes to the right, as shown in Fig. 3 for t > 0. This results in an increase of the spacing between the boxes, with D(t) being proportional to the density of the boxes.

If the entire gain occurred instantaneously at t = 0, all deaths would be postponed by the same amount; thus the spacing would again be dense and uniform after the first shift. In reality, the gain ΔL(t) increases gradually with t, and the resulting spacing depends on its rate of change

ΔL'(t) = dΔL(t)/dt.     (6)

To find the density of boxes, note that between t_i _+ ΔL(t_i_) and t_i+1 _+ ΔL(t_i+1_) there is one box. For small δt, this time interval is δt + ΔL'(t_i_)δt, and the density is 1 box/[δt + ΔL'(t_i_) δt]. At time t + ΔL(t), the ratio of the density of boxes after and before the intervention is therefore



and since D is proportional to the density one obtains

D(t+ΔL(t)) = D_ref_/(1 + ΔL'(t))     (8)

for t > 0. For a reduction of the exposure, ΔL(t) increases with t, ΔL'(t) is positive and D is less than D_ref_. For later reference, let us note that Eq.8 also holds for the case where the exposure increases and ΔL'(t) is negative. When the asymptotic gain ΔL∞ has been reached, ΔL'(t) vanishes and D(t) is again equal to the initial value D_ref_, because the birth rate has remained constant. But the mortality rate μ has decreased permanently, because the population size N has increased in proportion to the LE.

So far, we have assumed that all individuals have identical gains. In reality, there is a distribution of different individual gains. Averaging the quantity 1 + ΔL'(t) over this distribution, the ratio D_ref_/D(t) is replaced by its average over the individual gains. For the small changes involved in air pollution studies, the average of D_ref_/D(t) is very close to D_ref _divided by the average D(t).

The LE gain ΔL(T), after a time T following the intervention, can be obtained by integrating the measured data for D_ref_/D(t+ΔL(t)) of Eq.8



since ΔL(0) is of course zero. This is an integral equation, because ΔL appears on both sides. However, in practice one can approximate the result by integrating in steps, obtaining the gain at t_k+1 _from the one at t_k_



The ultimate gain ΔL∞ is the limit reached when T → ∞; in practice, the finite observation period yields only a lower bound (note that the integrand is positive-definite for a permanent pollution decrease), but a leveling off of the integrand would indicate that one is getting close to the ultimate gain. The units of ΔL(T) are the same as the ones chosen for t since the integrand is dimensionless. The result is the gain per person averaged over the study population.

The mortality μ(t), and hence the relative risk, can be obtained by dividing D(t) by the size of the respective populations according to Eq.3. Since the birthrate is constant, the population size N(t) increases with LE, ultimately reaching

N∞ = N_ref _(L_ref _+ ΔL∞)/L_ref _    (11)

when new stationary conditions are established (L_ref _being the LE before the intervention). Whereas D returns asymptotically to the initial value, the mortality rate μ is lower because people live longer and the population size has increased.

The exact time dependence of N(t) and μ(t) would require a more detailed calculation, because, during the transition to the new stationary state, different age groups increase differently; but in any case, N(t) is bounded by N_ref _and N∞. Since the population-average LE gain is short compared to L_ref _(at most a few months compared to about 75 years, see Eq.17 below), the change of the population size is entirely negligible in practice, considering the uncertainties of the data, and one can use the approximation

RR(t) = μ(t)/μ_ref _≈ D(t)/D_ref_.     (12)

Since the change in relative risk ΔRR(t) due to typical exposures is small compared to unity, one can further approximate Eq.9, with negligible error, by



Stepwise integration as in Eq.10 can be used, although the ΔL(t) on the right hand side can be neglected if T is relatively short, as shown by the example in the following Section.

To conclude this section, I emphasize that the key result is Eq.9 (or 13), which yields the LE change after an intervention as time integral of the observed death rate (or change of relative risk). As written, it is appropriate for the entire population, but with the obvious addition of a label x for age, it also holds for a constant age segment or for a birth cohort of age x, as shown in Appendix B [see Additional file 2].

## Results

### LE change for time series

In the LE framework, the mortality measured by typical TS studies corresponds to an intervention study that lasts only one day. Thus, the LE change is given by Eq.13 with T = 1 day. It represents the acute loss of life immediately after a pollution peak. Let us insert into Eq.13 the relative risk for 10 μg/m^3 ^of PM_10 _found in the analysis of the NMMAPS data for 90 cities in the USA, as recently revised to correct for the GAM problem [24]; it is ΔRR(0) = 0.0021 for the GLM version of the analysis (the t = 0 indicates that this is for the first day). The result is

-ΔL(1 day) = ΔRR(0) × 1 day = 0.0021 day     (14)

for acute mortality, 1 day at 10 μg/m^3 ^PM_10_. It would be almost twice as high for the original GAM estimate of ΔRR(0) = 0.0041. The minus sign indicates a loss for a risk increase.

Recently Schwartz [2] and Zeger et al [1] have succeeded in extending the exposure duration up to T = 60 days, measuring, in effect, the average relative risk ΔRR_av _corresponding to the average concentration Δc_av _of PM_10 _during the period T. They find that ΔRR_av_/Δc_av _increases with T at least up to 60 days, the longest for which their method could be used. For all-cause mortality, Schwartz found that ΔRR_av_/Δc_av _increased linearly with T and, at 60 days, was about twice that for one day. Fairly similar results have been found in other studies that have extended the observation window [10]. Since ΔRR_av _is the average from t = 0 to T of the relative risk ΔRR(t) at time t, the result of Schwartz implies that

ΔRR(t) = ΔRR(0) (1 + 2 t/60 days).     (15)

Inserting this into Eq.13 yields

- ΔL(T) = ΔRR(0) T (1 + T/60 days)     (16)

up to 60 days; it increases in linear plus quadratic fashion, reaching 0.0021 × 60 days × 2 = 0.25 days after 2 months. This is the population average LE loss per person. For sensitive subgroups, the loss is of course much higher, but at the present time not enough is known about individual sensitivities.

It is interesting to compare these numbers with the ultimate LE gain ΔL∞ achievable by a permanent reduction of PM_10_. That can be calculated on the basis of the cohort studies, such as the one by Pope et al [14], which are essentially steady state comparisons of the effects of different exposures. Several authors have published such calculations [4,15-18], based on the cohort study of Pope et al [14], with essentially the same result for the same long term relative risk. For example, Rabl [4] found

- ΔL∞ = 92 days     (17)

for lifetime exposure at 10 μg/m^3 ^of PM_10_. This is much larger than ΔL(1 day) = - 0.0021 days of Eq.14, for two reasons: the latter is for a single day of exposure, and it includes only acute effects. The contribution of acute mortality to the LE loss from chronic exposure will be addressed in the last section.

For O_3_, only acute mortality has been measured until now. The meta-analysis by the World Heath Organization [25] provides a ΔRR for all-cause mortality of 0.003 per 10 μg/m^3 ^increase in the daily maximum 8-hour mean O_3_. Analogous to Eq.14 the corresponding LE loss is ΔL(1 day)_acute _= - 0.003 days.

### LE loss per death

For acute mortality the LE loss per air pollution death can be obtained by dividing the LE loss of Eq.14 by ΔN_deaths_, the number of acute deaths that are attributable to a pollution peak. If one calculates the latter by multiplying the daily mortality by the relative risk, one finds for a peak of one day

ΔN_deaths- _= (0.01/365) × 0.0021 = 5.8E-8     (18)

for 10 μg/m^3 ^PM_10_, taking a typical mortality for Europe and North America of μ = 0.01 per yr per person together with ΔRR(0) = 0.0021 (note that ΔN_deaths-_, like ΔL, is normalized per person). I have added the subscript - to indicate that this is a lower bound because it corresponds to the observed deaths (i.e. the solid black line in Fig. 1). The corresponding upper bound for the loss per death is

ΔL(1 day)/death < 100 yr/death     (19)

independent of exposure, because both numerator and denominator are proportional to exposure. Since the real loss per death is certainly much smaller, the number of attributable deaths must be much larger than what is observable.

The attributable deaths are all the deaths that have been advanced by pollution (i.e. the thin dashed line in Fig. 1). Unfortunately, that is not known. If everybody's death is advanced somewhat, even if only by an undetectably small amount, ΔN_deaths _would be equal to (0.01/365). If only a fraction f_sens _of sensitive individuals is affected,

ΔN_deaths+ _= (0.01/365) × f_sens _    (20)

independent of exposure, and hence the lower bound is

ΔL(1 day)/death > 0.21 yr/f_sens _    (21)

for 10 μg/m^3 ^PM_10_, proportional to exposure.

### Models for the repair processes

Whereas all the results up to this point follow from the data, the rest of the paper invokes models of the action of pollution damage and is thus more speculative. A model is necessary to estimate how the mortality rate will change during an intervention study, beyond the period for which data are available (60 days for PM_10_, at most a few days for O_3 _and SO_2_).

Let us assume that the LE loss is proportional to the concentration c of the pollutant. Furthermore, past concentrations have less impact now because the body is able to repair some of the damage, an ability well documented in the case of ex-smokers [19-21]. To account for repair, it seems plausible to assume exponential decay for the effect of past exposures. If there is only a single time constant τ one obtains the following model for the LE loss at time t due to a sequence of concentrations {c(t')} between t_0 _and t



where c(t') is the concentration at time t' and k is a proportionality constant. The minus sign is introduced, because concentrations are positive and ΔL is a loss. A more realistic model contains several terms with different time constants, as described in Appendix C [see Additional file 3]. If the body could not recover, the time constant(s) would be infinite and the LE loss would depend only on the cumulative exposure, not on its distribution over time

Leksell & Rabl [15] reviewed the studies of ex-smokers, especially the one by Doll et al [20], one of the most comprehensive long term studies of smokers and ex-smokers. They found that the recovery can be approximated quite well by an exponential decay model with two time constants: a time constant of 1.5 years with weight 0.3 and one of 13 years with weight 0.7. Similar conclusions can be drawn from the data in USDHHS [19].

Applying time constants from smoking studies to air pollution entails uncertainties. For PM, the similarities in pollutant composition and in the nature of the health end points may be close enough for this purpose. Röösli et al [22] have analyzed the two available intervention studies that involve PM_10 _and found a time constant of 1.1 yr for the Utah steel mill intervention [7] and 9 yr for the intervention in Dublin county [9]. These values are consistent with those from smoking if one notes that the duration of the Utah intervention was only about one year, too short to allow the determination of longer time constants, and whereas the change in Dublin was permanent the study period of Clancy et al covered only six years. For other pollutants, such as O_3 _and SO_2_, the estimation of time constants is more problematic.

At this point I use a model with a single time constant, for the purpose of illustration. For a permanent step decrease Δc of the concentration the LE gain is

ΔL(t) = - k Δc τ [1 - exp(-t/τ)]     (23)

with t = time after decrease. The ultimate gain, for t →∞, is

ΔL∞ = - k Δc τ.     (24)

One could include an age dependence in ΔL(t) and k, although the available data do not show any significant variation with age [26].

Since some of the repair probably does not begin immediately, some of the LE gain is delayed relative to the model of Eq.23. A more realistic model would include a distribution p(λ) of different lags λ between exposure and LE change, replacing Eq.23 by



### Possible outcomes

**Figure 4:**
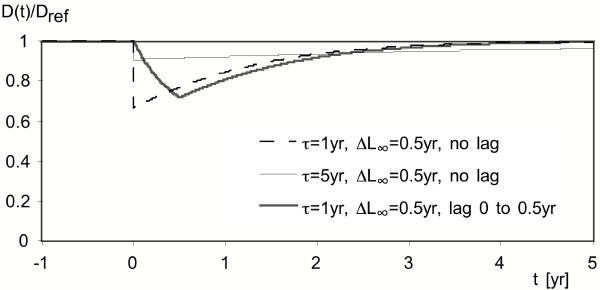
**Plot of deaths per time D(t), before and after intervention. **Results are shown for model with one time constant τ and ΔL∞ = 0.5 yr, assuming a stationary population.

Results are plotted in Fig. 4 for three models. For all of them, D(t) drops to a minimum soon after the intervention, but then increases again, becoming almost indistinguishable from the old values after several time constants. Without lag the initial drop is abrupt and has magnitude

D(0)/D_ref _= 1/(1 + ΔL'(0)) = 1/(1 - k Δc)     (26)

if no lag, regardless of the number of time constants in the model. With a distribution of lags, the drop is gradual, as shown by the thick gray line in Fig. 4 for which a uniform distribution of lags from 0 to 0.5 yr has been assumed arbitrarily. The area between the curve and the line D(t)/D_ref _= 1 is equal to ΔL∞.

## Discussion

### Relation between time series and cohort studies

Even though the designs of cohort and time series studies are very different, in particular with regard to the accounting for characteristics of individuals, the results should be consistent to the extent that the end point is comparable. That time series and cohort studies have a common ground has also been shown by Burnett et al [11], formulated in terms of relative risk (hazard function). Since the models of the Section Results yield the entire time dependence of the mortality after a change in exposure, they imply a relation between the results of TS and cohort studies.

In these models, the frail pool is implicit: it consists of the individuals who are going to die in the near future and whose deaths would be advanced by a pollution peak to the days following the peak. The LE change in these equations expresses the total effect of pollution on a stationary population, without distinguishing between acute effects from recent exposure and chronic effects from past exposure.

If the models are also valid for acute effects, they can be applied to a TS of fluctuations, because it does not matter whether concentrations increase or decrease: the models are linear and the exposure for each new day is added to the previous exposures. The concentrations are always positive, whether increasing or decreasing. The effects are symmetric between increases and decreases. A single peak of duration t is equivalent to the superposition of a permanent increase and a permanent decrease of equal magnitude t later. The change in mortality that would be found in a time series during the first day (or days) after a pollution peak is the change during the first day (or days, up to t) in Fig. 4.

As an example, consider the model of Eq.25 with one time constant τ and a distribution p(λ) of lags, applied to the entire population. With the approximations already made in Eq.13 for the relation between ΔRR(t) and ΔL'(t) for short times t one obtains

ΔRR(t)/Δc = k P(t)     (27)

with



With the TS result of ΔRR(0)/Δc = 2.1E-03 per 10 μg/m^3 ^PM_10_, Eq.14, this fixes the relation between k and P(t) at t = 1 day

k = 2.1E-03/P(1 day)     (29)

per 10 μg/m^3 ^PM_10_. On the other hand, combining Eqs.17 and 24 for the LE loss ΔL∞, one obtains

k τ = 0.23 yr     (30)

per 10 μg/m^3 ^of PM_10_. Assuming τ around 10 yr one finds k = 0.023 per 10 μg/m^3^. Thus the two estimates of k agree if P(1 day) = 0.0021/0.023 = 0.09 (i.e. if only 9% of the repair begins during the first day). Of course, the epidemiological estimates are quite uncertain, and the model for repair is speculative and crude, especially if it contains only one time constant. Nonetheless, it is encouraging that the two estimates of k are compatible.

### Contribution of acute mortality to LE loss from chronic exposure

The LE loss of Eq.14 is the acute mortality (i.e. the mortality during the first day of a one-day of exposure). For health impact assessments of pollutants for which only TS results are available, one needs to evaluate the acute impacts of successive one-day exposures. That is not simply the product of the one-day impact times the exposure duration, because past exposures are reduced by the repair capacity of the body. The LE loss of Eq.14 cannot be used directly, because it is for a single peak.

Let us split the LE change of Eq.25 into an acute contribution ΔL_ac _plus the rest, the acute term being the effect of each day's exposure during that same day. Assuming a one-time constant model for the acute term, one finds, analogous to the derivation of Eq.24, that the cumulative change resulting from an infinite series of one day exposures to Δc is

ΔL_ac_(∞) = - k_ac_Δc τ_ac_.     (31)

Since the change ΔL(1 day)= ΔRR(0) × 1 day (see Eq.14) from a single day is entirely due to acute effects, we can set k_ac _equal to k P(t) of Eq.27 and obtain

ΔL_ac_(∞) = ΔL(1 day) × τ_ac_/1 day.     (32)

For acute effects, a time constant around 1.5 year is plausible, because it corresponds to time constants for short term cardiovascular benefits found in smoking cessation studies [21]. That implies ΔL_ac_(∞) = -0.0021 days × 365 × 1.5 = -1.2 days, a little more than 1% of the total acute + long term ΔL∞ = -92 days of Eq.17.

In previous publications, a different approach to estimate the LE loss due to acute mortality has been used by the ExternE project series [3], namely calculating a number of deaths as product of baseline mortality rate and ΔRR, and multiplying it by assuming 6 months as LE loss per death. Whereas the resulting ratio of acute over total LE loss for PM_10 _was also about 1%, the method is not correct for several reasons:

• the total number of attributable deaths is not known, as explained in Section Results;

• the LE loss per death is not known;

• the calculation does not take into account the effect of repair, because it simply multiplies one-day impact by exposure duration.

Thus number of air pollution deaths, which was shown [4] to be meaningless for cohort studies (total air pollution mortality), is meaningless even for acute mortality. The approach of Eq.32 has the advantage of starting from a solid basis, namely the LE loss due to a single pollution peak; of course, it is also problematic, because it needs to invoke a repair model.

There are question marks about the models that I have assumed, quite apart from the number of time constants and the parameter values. In particular, the triggering of deaths among frail individuals during a pollution peak (via heart attacks that can shorten the life of a few individuals by a large amount) is different from the accumulation of damage among the general population (small incremental LE loss for many individuals). So the repair model may not be correct for all acute effects, and the symmetry between increases and decreases of exposure may be only approximate. In that case, the model(s) for LE change as function of exposure would have to be modified by an explicit model for the frail pool.

## Conclusion

By formulating the analysis of air pollution mortality in terms of LE (life expectancy) rather than mortality risk, one obtains a unified framework for time series studies, intervention studies and cohort studies. TS studies measure the instantaneous time derivative of LE changes due to pollution. One of the advantages of this approach is that it yields as rigorous model-independent result the LE change after a pollution peak or after an intervention as an integral of the observed mortality rates. However, the estimation of the number of deaths attributable to air pollution is problematic and so is the LE loss per air pollution death.

The relation between the results of the different study types depends on the processes by which the body repairs air pollution damage. Using plausible models for the repair processes, one finds that the mortality rates change most strongly in the initial period after the intervention, thereafter returning to a level close to the original, even though the population has obtained a permanent LE gain. The time scale depends on the time constant(s) of the repair processes. Unfortunately, not enough is known about repair processes at the present time to allow more specific conclusions.

With the assumed repair models, one finds that the results of TS studies are consistent with the ultimate LE change due to a permanent exposure change, as determined by cohort studies. This raises the interesting possibility of using repair models to estimate the LE gain achievable by a permanent reduction in O_3 _exposure, a pollutant for which a significant effect has been identified so far only by TS and not by cohort studies.

## Abbreviations and symbols

c = concentration of pollutant;

Δc = concentration change;

D = death rate of population or population segment, absolute number [deaths/time];

D_ref _= reference death rate of population, in the absence of an intervention [deaths/time];

k = proportionality constant for relation between Δc and ΔL;

LE = life expectancy;

L = life expectancy;

L_ref _= reference life expectancy, in the absence of an intervention;

L(x_0_) = remaining life expectancy (survival time);

ΔL = change in life expectancy [yr/person], positive for a gain;

ΔL(t) = change in life expectancy at time t after a permanent decrease of concentration;

ΔL∞ = ultimate change in life expectancy (long after a permanent decrease of concentration);

N = population size;

RR = relative risk;

S(x_0_, x) = survival function = fraction of birth cohort of initial age x_0 _that survives to age x;

t = time;

TS = time series

w_i _= weighting factors of different time constant in repair model;

x = age;

λ = lag time of repair model;

μ = mortality rate = death rate/population size [deaths/time per person];

μ_ref _= reference mortality rate;

τ = time constant of repair model;

## Competing interests

The author(s) declares that he has no competing interests.

## Supplementary Material

Additional File 1 Appendix A. Relation between age-specific mortality and life expectancy. Click here for file

Additional File 2 Appendix B. Change due to intervention, by age group. Click here for file

Additional File 3 Appendix C. Repair model with several time constants. Click here for file
