# Supplementary material for: Analysis of air pollution mortality in terms of life expectancy changes: relation between time series, intervention, and cohort studies
Source: Environ Health. 2006 Feb 1;5:1. doi: 10.1186/1476-069X-5-1 (PMC1373624; doi:10.1186/1476-069X-5-1)
Supplement: Additional File 1 — Appendix A. Relation between age-specific mortality and life expectancy. [file 1476-069X-5-1-S1.doc]

**Appendix A. Relation between age-specific mortality and life expectancy**

The survival function S_μ_(x_0_, x) is determined as the solution of the differential equation

dS_μ_(x_0_,x) = - S_μ_(x_0_,x) μ(x) dx, (A.1)

with the boundary condition S_μ_(x_0_,x_0_) = 1. The solution is

S_μ_(x_0_,x) = exp[- μ(x’) dx’]. (A.2)

Since the probability of a cohort member to survive to age x and die between x and x+Δx is S_μ_(x_0_,x) μ(x) Δx, the remaining life expectancy L(x_0_), also known as expected survival time, of this cohort is obtained by integrating the age x times this probability over the entire cohort

L(x_0_) = x S_μ_(x_0_,x) μ(x) dx. (A.3)

Using Eq.A1 one sees that L(x_0_) is the area under the survival function, bounded by the x-axis and the y-axis at x=x_0_. Integrating by parts one obtains

L(x_0_) = S_μ_(x_0_,x) dx. (A.4)

If μ(x) is given, S_μ_(x_0_,x) and L(x_0_) are uniquely determined. Vice versa, by the following steps one can show that L(x_0_) determines μ(x). The derivative of L(x_0_) is

, (A.5)

and with Eq.A1 this becomes

= -1 + μ(x_0_) L(x_0_), (A.6)

from which one obtains

. (A.7)

as an explicit function of L(x).
