# Supplementary material for: Analysis of air pollution mortality in terms of life expectancy changes: relation between time series, intervention, and cohort studies
Source: Environ Health. 2006 Feb 1;5:1. doi: 10.1186/1476-069X-5-1 (PMC1373624; doi:10.1186/1476-069X-5-1)
Supplement: Additional File 2 — Appendix B. Change due to intervention, by age group. [file 1476-069X-5-1-S2.doc]

**Appendix B. Change due to intervention, by age group**

The arguments of Section Methods can also be used for a group of a specified initial age, either a birth cohort that has age x_0_ at the time of the intervention or a “constant age segment”, i.e. a population segment the age of which is held fixed. In the case of the birth cohort the death rate D_ref_(x_0_,t) in the absence of the intervention also changes with time, and one needs to consider the relation between D(x_0_,t) and D_ref_(x_0_,t). Generalizing Fig.3 to a situation where D_ref_(x_0_,t) varies with time, one readily finds

D(x_0_,t+ΔL(x_0_,t)) = D_ref_(x_0_,t)/(1 + ΔL’(x_0_,t)) (B.1)

for t > 0 (now ΔL’(x_0_,t) designates the partial derivative of ΔL(x_0_,t) with respect to t). The size of a birth cohort is equal to the birth rate times the fraction S_μ_(0,x,t) that survives to age x

S_μ_(0,x,t) = exp[-μ(x’,t) dx’] (B.2)

with x = x_0_ + t. Therefore the age-specific mortality rate and the relative risk RR(x,t) are determined by

RR(x,t) = μ(x,t)/μ_ref_(x,t) = [D(x,t)/D_ref_(x,t)]/[S_μ_(0,x,t)/S_ref_(0,x,t)]. (B.3)

This is an integral equation since S_μ_(0,x,t) involves an integral of μ(x,t). However, as first approximation one can set

μ(x,t)/μ_ref_(x,t) ≈ D(x,t)/D_ref_(x,t) (B.4)

for the small changes encountered in practice. Then one could improve the approximation by iterations, if desired. Thus the second approximation involves inserting μ(x,t) = μ_ref_(x,t) D(x,t)/D_ref_(x,t) into Eq.B.3 and using it to calculate an improved estimate of S_μ_(0,x,t). That the corrections are very small can easily be verified by comparing S_ref_(0,x,t) with S_μ_(0,x,t) in the limit of very large t when S_μ_(0,x,t) approaches the steady state limit corresponding to the ultimate LE gain ΔL_∞_. As an example let us take a relative risk of 1.06 for a concentration change of 10 μg/m^3^ of PM_2.5_ [12, for the average concentration 1979-2000, in their Table 2]. It implies an ultimate LE gain of 0.4 yr for a permanent decrease of PM_2.5_ by 10 μg/m^3^. One finds that the corresponding difference between S_ref_(0,x,t) and S_μ_(0,x,t) is at most a small fraction of a percent for ages below 50. Around 70 to 80 the relative difference becomes larger, up to a few percent, but even that implies only a small correction for the difference between μ(x,t)/μ_ref_(x,t) and D(x,t)/D_ref_(x,t), a correction that could be taken into account adequately by one iteration if necessary.
