# Supplementary material for: Analysis of air pollution mortality in terms of life expectancy changes: relation between time series, intervention, and cohort studies
Source: Environ Health. 2006 Feb 1;5:1. doi: 10.1186/1476-069X-5-1 (PMC1373624; doi:10.1186/1476-069X-5-1)
Supplement: Additional File 3 — Appendix C. Repair model with several time constants. [file 1476-069X-5-1-S3.doc]

**Appendix C. Repair model with several time constants**

Processes with different time constants can be incorporated by making the replacement

exp[-(t-t’)/τ)] → w_i_ exp[-(t-t’)/τ_i_)] (C.1)

(with the sum of the weights w_i_ equal to unity) in the equations for the models. Leksell & Rabl [13] reviewed the studies of ex-smokers, especially the one by Doll et al [15], one of the most comprehensive long term study of smokers and ex-smokers. They found that the recovery can be approximated quite well by an exponential decay model with two time constants: a time constant of 1.5 years with weight 0.3 and one of 13 years with weight 0.7. With this model the LE gain ΔL(t) after the cessation of smoking is

ΔL(t) = - ΔL_∞_ {1 – [w_1_ τ_1_ exp(-t/τ_1_) + w_2_ τ_2_ exp(-t/τ_2_)]/(w_1_ τ_1_+ w_2_ τ_2_)} (C.2)

where w_1_ =0.7, τ_1_ = 13 yr, w_2_ = 0.3, τ_2_ = 1.5 yr, and ΔL_∞_ is the ultimate gain.
